# Predicting phenotypes of elderly from resting state fMRI

**DOI:** 10.21203/rs.3.rs-3201603/v1

**Published:** 2023-08-07

**Authors:** Barbara Verovnik, Stefan Hajduk, Marc Van Hulle

**Affiliations:** University of Ljubljana; KU Leuven; KU Leuven

**Keywords:** Resting-state fMRI, Functional connectomes, Predictive modeling, Classification, Aging

## Abstract

Machine learning techniques are increasingly embraced in neuroimaging studies of healthy and diseased human brains. They have been used successfully in predicting phenotypes, or even clinical outcomes, and in turning functional connectome metrics into phenotype biomarkers of both healthy individuals and patients. In this study, we used functional connectivity characteristics based on resting state functional magnetic resonance imaging data to accurately classify healthy elderly in terms of their phenotype status. Additionally, as the functional connections that contribute to the classification can be identified, we can draw inferences about the network that is predictive of the investigated phenotypes. Our proposed pipeline for phenotype classification can be expanded to other phenotypes (cognitive, psychological, clinical) and possibly be used to shed light on the modifiable risk and protective factors in normative and pathological brain aging.

## Introduction

Among the neuroimaging techniques, functional magnetic resonance imaging (fMRI) has advanced most our current understanding of healthy and diseased human brains^1^. Traditionally, fMRI studies focus on general patterns of brain activity across individuals^2^. It is without doubt that these studies provided valuable insights into a variety of brain functions, however, by design, they demote the effect of within-group heterogeneity^2^, even though the information they offer could still be key to understanding the neurobiological underpinnings of brain function. Additionally, as traditional univariate analysis is limited to charting the neural correlates of the observed behavior, it cannot inform us about future developments^3^. In order to address these limitations, a novel approach is needed^3,4^. Machine learning techniques used to integrate multiple brain regions have been successful in predicting phenotypes or even clinical outcomes^5^ and in turning functional connectome metrics into phenotype biomarkers of both healthy individuals and patients^4^. As the machine learning approach benefits from large-scale datasets^4^, it relies on the aggregation and sharing of neuroimaging data.

Resting-state functional connectivity (RSFC) reflects the synchrony in BOLD signals between brain regions^6^ and can be used to identify large-scale brain networks^7^ as well as to predict individual differences in behavior^2^. Given the versatility in applications, there is a notable interest in estimating subject-specific brain networks^8,9^ using machine learning approaches^3,5^, especially in connection with mental traits and behavioral dispositions. Several studies suggest that not only age and gender but also complex mental traits such as intelligence^5^, attention^10^ and personality factors^5^ could be predicted from brain imaging data. Recent years have witnessed an increasing interest in modifiable risk factors and how they influence normative and pathological aging (e.g. obesity, cardiovascular factors, social and psychological factors, environmental factors)^11^. Those risk factors are known to affect functional and structural neuroimaging^12^ and could be considered when developing models aimed at predicting which individuals are at risk of cognitive decline.

Brain age is perhaps the most researched and established proxy of a person’s age. Predicted from structural and functional imaging^5,13,14^, it is clear that it reflects more than just biological age as it accounts for brain damage^15^, cognitive impairments^16^ and neurodegeneration^14,17^. Brain age delta (the difference between brain-based predicted age and biological age) captures the deviations from population norm and is often used as a marker of brain aging^18^. A higher brain age delta is shown to be correlated with adverse health behaviors such as alcohol consumption, smoking, high blood pressure, diabetes, worse physical fitness, lower fluid intelligence and cognitive reserve^13,16^. Additionally, multiple studies have shown a higher brain age delta in pathological aging: patients with cognitive impairments and Alzheimer’s disease exhibit a higher brain age delta than heathy controls^14,17^. Biological sex has proven harder to accurately classify compared to age. Even though, there are distinctive structural and functional differences between the sexes, there is also substantial overlap in functional connectivity, such as in terms of the small-world network characteristic^19^. Studies using fMRI data achieved a classification accuracy for biological sex of approx. 80%^20,21^.

A person’s handedness could be predictive of hemispheric asymmetry and impact not only motor processing but possibly also cognitive, language, sensory and emotional processing^22^. Additionally, in some reports, approx. 70–80 % of left-handed people have been shown to have typical, left hemisphere language dominance but there are also 20–30 % of left handed people who have language processing that is atypical and distributed across the hemispheres, contributing to the observed variability in brain measures^23^. Because of such differences, left-handed individuals are often excluded from neuroimaging studies. Studies looking into handedness as the only proxy for hemispheric asymmetry are sparse^24^, as handedness has a complex relationship with lateralization of different functions (e.g. language)^25^. Finding a brain proxy for handedness could enable the inclusion of left-handed people in neuroimaging studies. Chormai et al.^24^ managed to classify handedness with 72 % accuracy using functional connectivity. They also tried to interpret the model and looked at the features that were most important for handedness prediction. It appeared that motor and somatosensory networks were most predictive of handedness, however, other networks were also involved (language processing, social interaction, arithmetic, and the default mode network).

There is also some evidence of brain correlates of other demographic factors. For example, education has been widely used for assessing cognitive reserve (neural resources dependent on genetic and environmental factors that attenuate age-related or pathological cognitive decline^26^), which in turn has been associated with functional connectivity of attention and executive networks^27,28^. There have also been some efforts in classifying other phenotypes based on functional connectivity such as personality^18^ and intelligence^29^.

The cited studies show that predictive modeling and machine learning approaches can be used for classifying and predicting phenotypes based on fMRI data^3^. However, predictive models rarely inform us about which features are important for classification or prediction. One of the biggest challenges, in particular when relying on machine learning, is interpretability and explainability^30^. Providing interpretable models broadens the knowledge about the underlying reasons why groups/classes can be distinguished which in turn can be used for explaining the relevance of these differences in terms of diseases and phenotyping.

The goal of this study is to identify a set of functional connectivity characteristics based on RS fMRI data that would classify individuals in terms of their phenotype status. The interpretability of the model is part of this goal, which we achieved by tracing classifier accuracy back to its most contributing connectivity features. This goes beyond the accuracy and ROC analysis most biomedical modeling algorithms are limited to. Interpreting these features enables us to draw inferences about the functional connectivity characteristics that are predictive of the investigated phenotype classes and to uncover differences between groups of healthy elderly.

## Results

**Figure 1:**
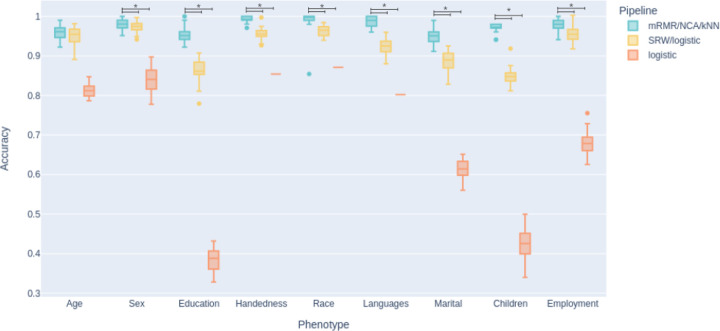
Classification accuracies achieved with three pipelines and their statistical comparison. Horizontal lines with asterisks indicating statistical significance (two-sided t-test, p<0.05). *Note.* mRMR = minimum Redundancy Maximum Relevance; NCA = Neighborhood Component Analysis; kNN = K-nearest neighbor; SRW = sequential replacement wrapper; logistic = logistic regression.

When comparing the proposed phenotype classification pipeline, which comprises minimum Redundancy Maximum Relevance feature selection/Neighborhood Component Analysis/k-nearest neighbor classification (mRMR/NCA/kNN), with logistic regression, as proposed by Dadi et al. [31], and its extension, the sequential replacement wrapper (SRW)/logistic regression pipeline, the mean cross-validated accuracy for all phenotypes except age was higher for the mRMR/NCA/kNN pipeline. Fig. 1 shows the classification accuracies of the three pipelines for all phenotypes with the significant differences marked.

### Age

After initial feature selection with mRMR, NCA and k-NN tuning for classification accuracy was performed. The best combination was 3 NCA components, 3-NN with 240 best features selected with the mRMR filter. The mean cross-validated accuracy for the classifier was 0.96 (σ = 0.02). When applying the SRW/logistic regression pipeline, 37 features were selected yielding a mean cross-validated classification accuracy of 0.96 (σ = 0.02). The lowest classification accuracy was achieved when using logistic regression only (0.81, σ = 0.01). Significant connections, according to the permutation feature importance technique^31^, for the classification of age using the proposed pipeline are listed in Table 1.

### Biological sex

Mean cross-validated accuracy for the classification of biological sex was 0.98 (σ = 0.01) when using 350 best features, 2 NCA components and 1-NN. When applying the SRW/logistic regression pipeline, 53 features were selected for which the mean cross-validation accuracy was 0.97 (σ = 0.02); logistic regression yielded the worse classification accuracy, 0.84 (σ = 0.04). The significant connections for the classification of biological sex using proposed pipeline are listed in Table 1.

### Education

In the case of education, the best accuracy was for 530 features, 3 NCA components and 4-NN, yielding a mean cross-validation accuracy of 0.95 (σ = 0.01). The SRW/logistic regression pipeline yielded a mean cross-validation accuracy of 0.86 (σ = 0.04) for 78 best features; logistic regression again returned the lowest accuracy of 0.39 (σ = 0.03). All significant DiFuMo ROIs connections using the proposed pipeline are listed in Table 2.

### Handedness

The average classification accuracy of all cross-validation folds for handedness was 0.99 (σ = 0.01), using 350 best features, 3 NCA components and 1-NN. When applying the SRW/logistic regression pipeline, 33 features were selected for which the mean cross-validation accuracy was 0.96 (σ = 0.02). Logistic regression achieved an accuracy of 0.85 (σ = 0.00). Significant connections for the proposed pipeline are listed in Table 3.

### Race

Six subjects had missing values and were therefore discarded, hence, the pipelines were applied to 335 subjects. The mean cross-validated accuracy for the classification of race was 0.99 (σ = 0.01) when using the proposed pipeline with 600 selected features, 2 NCA components and 1-NN. The SRW/logistic regression pipeline yielded a mean cross-validation accuracy of 0.96 (σ = 0.01) with 34 features selected. When applying logistic regression only, the mean cross-validation accuracy was 0.87 (σ = 0.01). Table 3 lists the significant connections using the proposed pipeline.

### Number of languages spoken

The same six subjects who had missing values for race, also had missing values for the number of languages and were therefore discarded, resulting in 335 subjects. The best combination of classification performance for the proposed pipeline was for 500 best features, 3 NCA components and 1-NN, yielding a mean cross-validation accuracy of 0.95 (σ = 0.02). The mean cross-validation accuracy of the SRW/logistic regression pipeline was 0.92 (σ = 0.02) using 26 features. Logistic regression yielded a mean cross-validated accuracy of .87 (σ = 0.00). One significant connection for the proposed pipeline is listed in Table 3.

The modeling of the last three phenotypes (marital status, number of children and employment status) was done on 339 subjects as two subjects had missing values.

### Marital status

Using the proposed pipeline, the best mean cross-validation accuracy of 0.95 (σ = 0.02) was achieved with 400 features, 3 NCA components and 4-NN. Applying the SRW/logistic regression pipeline resulted in 55 features with mean cross-validation accuracy of 0.89 (σ = 0.03). When applying logistic regression only, the classification accuracy was the lowest, 0.60 (σ = 0.03). Table 4 lists the significant connections for the proposed pipeline.

### Number of children

The best parameter combination of the proposed pipeline was for 400 features, 4 NCA components and 4-NN, yielding a mean cross-validation accuracy of 0.97 (σ = 0.02). Using the SRW/logistic regression pipeline with 73 features yielded a mean cross-validated accuracy of 0.85 (σ = 0.03). Logistic regression performed the worst as the mean cross-validated accuracy was 0.42 (σ = 0.04). All significant connections identified using the proposed pipeline are listed in Table 4.

### Employment status

The average classification accuracy of all cross-validation folds for employment status was 0.97 (σ = 0.02), using 680 best features, 3 NCA components and 4-NN for the proposed pipeline. Using SRW/logistic regression with 57 features, a mean cross-validated accuracy of 0.95 (σ = 0.02) was obtained. The lowest classification accuracy achieved was with logistic regression, 0.67 (σ = 0.03). Table 5 reports significant connections using the proposed pipeline.

## Discussion

We sat out to develop a machine learning pipeline that can accurately classify several demographic phenotypes and that supports the identification and interpretation of the most influential brain connections that underlie the classification. In our study, the combination of mRMR filtering, NCA and k-NN classification yielded superior accuracies for all demographic phenotypes, when using only functional connectivity measures, compared to logistic regression^32^ and the combination of sequential replacement wrapper and logistic regression.

When it comes to classifying individuals by age, we achieved a superior accuracy compared to previous studies^13–15^; note that all 3 compared pipelines performed similarly for this phenotype. This is in part due to our normative sample, where we expect smaller differences between chronological and brain age. We also identified the most significant connections for classifying individuals by age and found that the intraconnectivity of the dorsal attention (DAN), frontoparietal control (FPCN), visual (VIS) and somatomotor (SOM) networks drives the classification. Additionally, the interconnectivity between DAN, FPCN, VIS, SOM and default mode network (DMN) is also contributing to the classification (Table 1). This is in line with previous literature that has implicated those networks in aging^33–35^. There is a number of studies showing decreases in functional connectivity in DMN with aging as well as in pathological cognitive decline (mild cognitive impairment, Alzheimer’s disease)^33^. Our study found the functional connectivity between SOM and DMN to be important, which has also been shown to be present in aging, and could indicate that motor impairments often occur with advancing age^36^. Age-related deficits in cognitive control and attention have since long been observed^35,37^. Neuroimaging studies have corroborated these behavioral findings, showing less engagement of the frontoparietal network when individuals perform a cognitive control task^38^ (e.g. Stroop task^35^) and that at rest this network exhibits reduced intraconnectivity^38,39^. Not only that, age-related decreases in DAN have also been observed^40^, which is consistent with behavioral observations^37^. Additionally, our pipeline identified the SOM network as being important when classifying individuals based on age. Previous research has identified changes to primary RSFC networks in aging individuals (VIS and SOM)^41^. These findings are not surprising as visual and auditory deficits are common in old age, affecting perception and eventually cognition^39^. Other studies have also found within-connectivity decline in of the SOM network^34,39^. However, these findings are less clear as Tomasi and Volkow^40^ have shown increases in intraconnectivity of somatosensory and motor networks during aging. This heterogeneity in findings might be in part due to the larger interindividual differences that are observed during normal aging^41^.

The pipeline proposed in this study also performed superior to other studies when classifying individuals based on biological sex. The average classification accuracy was 98% compared to previous studies that archived accuracies in the 80s^20,21^. For the classification of sex, cerebellar ROI, DMN, salience/ventral attention network (VAN), DAN, VIS, FPCN, and limbic network were important (Table 1). The previous studies implicate that DMN, FPCN, DAN and VAN play a role in sexual dimorphism^40,42,43^. In our study, cerebellum was also shown to be implicated. The study of the cerebellum is often neglected. However, some research has also looked into it. Structural sex differences were observed, specifically larger cerebella in males^44^. When it comes to functional connectivity, women experienced higher functional connectivity in the cerebellum than men^45^. Gao and collegues^46^ also showed that, in addition to cerebral networks, the inclusion of the cerebellum network improves sex prediction by more them 2%, which hints at the importance for sexual dimorphism.

Education, usually considered when assessing cognitive reserve in aging research, has been connected with alterations in functional connectivity of the attention networks (DAN, VAN) and of FPCN^27,28^. That was also the case with the proposed pipeline as the most influential features involved a change in functional connectivity in the DAN, VAN and FPC, as well as the DMN, VIS and SOM networks (Table 2). Additionally, occupational attainment is also considered a cognitive reserve proxy^47^. Our results show intraconnectivity in FPCN, DMN and VAN to be associated with classification of late life employment (Table 5). A study by Franzmeier and collegues^48^ has linked cognitive reserve with FC of frontal areas (FPCN) and DMN and DAN, respectively.

The accuracy of handedness classification was also superior to that of a previous study^24^, showing that functional connectivity can be used to reliably classify an individual’s dominant hand. This could be especially useful in future studies, where the sample will not need to be restricted to right-handed individuals. Having information on an individual’s handedness from the brain connectivity could be a more reliable way of assessing this phenotype than questionnaires and could be entered into the analysis as a covariate to control for it. Our study showed that connectivity between somatomotor, SN, DAN and FPCN drive the classification (Table 3). This is in line with a previous study, showing that attention- and fronto-temporal networks are involved in handedness^49^.

The other phenotypes we considered are far less researched, and literature showing differences in functional connectivity is scarce. For example, differences in RSFC of DMN between older African Americans and Caucasians have been observed; African Americans had lower connectivity between MTL, precuneus, temporal pole and inferior temporal cortex^50^. Our study did not find differences in DMN but showed differences in the interconnectivity of DAN, salience- and visual networks between White Caucasian African American and Asian individuals (Table 3).

Studies investigating bilingualism showed differences in DMN and FPCN, which is consistent with behavioral studies showing changes to cognitive control/executive functions in individuals with fluency in two languages^51^. Our study, on the other hand, identified interconnectivity of the VIS network to be relevant for the accurate classification of the 3 groups based on number of languages spoken (Table 3). There is also much to be considered when assessing the effect of spoken languages, for example proficiency, age of acquisition, which could lead to a great deal of variability unaccounted for in our study.

A high classification accuracy was achieved for marital status- and number of children phenotypes (95% and 97%, respectively) with quite a few important features spanning various networks. Given that previous literature is virtually non-existent, it is hard to connect our findings. A study investigating social relationships and networks showed increased RSFC in FPCN with better social support^52^. Both phenotypes identified features from FPCN as important for classification, corroborating our findings (Table 4). Additionally, studies of loneliness stress the role of visual attention, limbic networks^53^ and DMN^53^. Our analysis showed important features in VIS, SOM networks, DAN, DMN, VAN and limbic network, which is consistent with the studies cited. However, it is important to mention that these phenotypes exhibit extensive variability and, therefore, multiple networks could be involved.

## Conclusion

This study has shown that demographic variables of elderly can be accurately predicted from resting state functional connectivity of fMRI data. The proposed mRMR/NCA/kNN classification pipeline showed superior accuracy, especially compared to logistic regression, which was previously identified as yielding the best performance among several other pipelines^32^. Further improvements are imaginable in terms of the used machine learning and filtering approach and worth perusing in the future. With the use of permutation feature importance, we identified significant connections that provided interpretability and explainability of the obtained results.

Our results also show great potential for predicting various other phenotypes such as cognitive, psychological or even clinical ones, with applications for healthy individuals to better understand normative processes in the brain as well as a variety of neurological diseases. RS-fMRI is particularly suitable paradigm for collecting functional connectivity data in the cognitively impaired and elderly as it avoids possible issues with task-based fMRI or with excessive head movements when longer sequences are considered.

With further developments to our pipeline, we intend to pursue other phenotypes, especially those that can be used to identify latent groups of elderly subjects at risk of cognitive decline. This is an important goal, as the early identification of at-risk individuals would enable early interventions such as programs aimed at slowing down the progression of cognitive decline and possible AD. With the inclusion of risk, protective and especially modifiable risk factors, additional information can be extracted to aid in developing effective early interventions for at-risk individuals.

## Methods

### Participants

The data used for this study originates from the Human Connectome Project – Aging (HCP-A). The subjects included in this study are considered a ‘typical’ population regarding health, representative of gender, race, ethnicity, and socio-economic status of the United States for the age range 36 to 100+ years. For details regarding the used inclusion and exclusion criteria and behavioral testing, we refer to the literature^54^. The HCP-A obtained approval from Institutional Review Board, and all participants provided written, informed consent. Statements on compliance with ethical standards for this study are provided in the Ethics declaration section. The HCP-A database currently boasts structural MR imaging, diffusion tensor imaging, task and resting-state fMRI, and behavioral data (cognitive tests, personality, lifestyle questionnaires) of 724 individuals, 341 of which are 60 years or older and included in our study; their average age was 74.56 years (SD = 9.59). The sample consisted of 156 men (45.7 %) and 185 women (54.3 %). Their education ranged between 7 and 21 years with average of 17.55 years (SD = 2.21). The majority were identified as White Caucasians (N = 292, 85.6 %), 26 as African American (7.6 %), 17 as Asian (5.0 %) and 6 as other (1.8 %). Handedness was determined with the Edinburgh Handedness Inventory^55^, using the laterality quotient, with scores above 40 deemed right-handed and below −40 left-handed. Individuals with scores between 35 and −35 were classified as mixed handed. In the sample, 290 (85.0 %) participants were identified as right-handed, 13 (3.8 %) as left-handed and 38 participants (11.2 %) as mixed-handed. Demographical data were categorized into phenotypes and used for classification. For this study, nine phenotypes were used. Age, sex and employment status were the only binary phenotypes. All other variables were divided into three classes. The phenotypes and their classification are listed in Table 6.

### Resting state fMRI data

Resting-state fMRI (RS fMRI) data were acquired with a Prisma 3T using a 2D multiband gradient-recalled echo (GRE) echo-planar imaging (EPI) sequence (MB8, TR/TE = 800/37 ms, flip angle = 52°) and 2.0 mm isotropic voxels covering the whole brain (72 oblique-axial slices). The data was acquired in 4 runs, in AP/PA phase encoding directions interchangeably. Participants were instructed to fixate on a white crosshair on a black background to mitigate any movement artefacts. Each run collected 488 frames, representing 26 min of RS fMRI in total for each participant. More information about the MRI acquisition can be found elsewhere^56^.

### Preprocessing

The imaging data we downloaded from the HCP servers was already preprocessed as follows. The RS fMRI files were aligned across subjects using nonlinear volume registration. The data was then passed through the HCP Functional Preprocessing for resting-state scans, applying the fMRIVolume Pipeline and HCP version of FSL’s FIX tool for denoising of fMRI data using spatial ICA^57^. A detailed description of the HCP acquisition and preprocessing pipelines can be found elsewhere^57^.

### Functional connectome-classification pipeline

**Figure 2:**
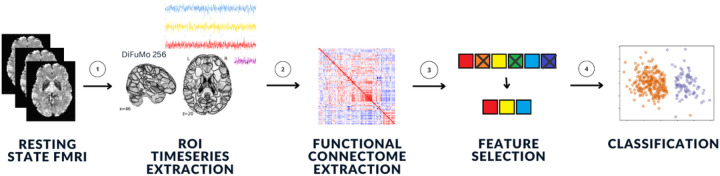
Classification pipeline consisting of 4 main steps (see text).

Functional data was further processed and analyzed in 4 steps (Fig. 2): 1) parcellate the brain regions of interest (ROIs) from resting-state fMRI images, 2) extract the functional signals (i.e. timeseries) and create the functional connectome, 3) perform feature selection using a filter approach, and 4) classify the phenotypes from connectivity features extracted from previous steps.

We adopted the functional connectome-based predictive methods pipeline proposed by Dadi et al.^32^ with brain regions of interest (ROIs) parceled using the Dictionary of Functional Modes for brain imaging (DiFuMo) multidimensional atlas^58^. For our study, we used the 256-dimensional atlas of functional modes to extract functional signals (i.e. timeseries), thereby adapting the scripts available from github.com/KamalakerDadi. This dimensionality is similar to the 300 nodes considered optimal number for predicting phenotypes from functional connectomes^58^. After extracting the timeseries, the data of 341 participants was used to build the connectomes. Connectivity parametrization was done using the tangent space of covariance matrices, as it was shown to outperform other types of parameterization^32^. The functional connectome was then vectorized, using the lower triangular part of the matrix, including its diagonal, for classification. Next, a minimum Redundancy Maximum Relevance (mRMR) filter was used for feature selection, providing a ranking of features from best to worst performing. The final step involved the prediction of the phenotypic status from connectivity features extracted from previous steps. First, a dimensionality reduction was performed using a Neighborhood Component Analysis (NCA)^59^, a supervised subspace method that finds the linear transformation that best separates data with different labels. Then, a K-nearest neighbor (k-NN) classifier was used for classification of the test data into phenotype classes. For each phenotype, NCA and k-NN parameters were tuned for classification accuracy. In previous work, Dadi et al.^32^ achieved the best performance using (l2-regularized) logistic regression. Thus, as a comparison, we used logistic regression but also implemented a possible improvement by combining the former with a sequential replacement wrapper (SRW), a combination of forward- (starting with 1 feature and adding features to the model subsequently only if they improve the performance) and backward feature selection (when a new feature is added, it tries to remove features which are already included only if they improve the performance). In order to figure out which features were significant for the classification of a particular phenotype, we implemented the permutation feature importance technique^31^. It gauges model's behavior by measuring the increase in the model’s prediction error after permutation of the feature values and, when done for all features, it can be used to rank their relative predictive power (using the permutation feature importance score, PFI score). In our study, PFI score represents the change in model’s classification accuracy after permutation of a given feature. In the results we report only those features that have a positive PFI score when two times standard deviation is subtracted from the PFI score.

### Cross-validation

We performed stratified and shuffled 20-fold cross-validation using a 70/30 split between training and testing data for all analyses. The folds were stratified to ensure that the same ratio of classes is included in the training and test sets. The permutation feature importance technique needs data on which the model was not trained. Hence, a 70/30 split before model construction was made with the 70 split used for model training, the 30 split for permutation feature importance assessment. The accuracy of the classification model is averaged over all splits, where 1 is a perfect prediction and 0.5 is chance level for binary classification or 0.33 for ternary classification.

### Technical implementation

In order to extract the representative timeseries, and to build the connectivity measures, we relied on the open-source Python 2.7 package Nilearn v0.9.2^60^. Machine-learning methods used for classification and cross-validation were implemented with scikit-learn v1.1.2^61^. For the visualization of brain connectivity, we relied on Nilearn matplotlib^62^ and plotly^63^. Documentation and Jupyter notebooks generated for this study are available 10.5281/zenodo.7862916.

## Figures and Tables

**Table 1.** Significant functional connections for age and biological sex phenotypes.

Phenotype	Feature	PFI score (std)	DiFuMo atlas ROIs
Age	1652	0.080 (0.025)	Inferior frontal gyrus LH - Inferior frontal gyrus LH (FPCN – FPCN)
	29413	0.062 (0.027)	Postcentral sulcus middle RH - Superior Parietal Lobule(HO) (SOM – DAN)
	23435	0.061 (0.027)	Parieto-occipital sulcus posterior - Parieto-occipital sulcus posterior (VIS – VIS)
	423	0.050 (0.018)	Angular gyrus superior RH - Lateral Occipital Cortex(HO) (FPCN – DAN)
	7236	0.035 (0.017)	Lateral occipital cortex superior - Superior occipital gyrus RH (DAN – DAN)
	10013	0.035 (0.017)	Central and postcentral sulci superior - Superior Parietal Lobule(HO) (SOM – DAN)
	3702	0.035 (0.016)	Heschl’s gyrus - Frontal operculum RH (SOM – SOM)
	30363	0.015 (0.005)	Postcentral Gyrus(HO) - Dorsomedial prefrontal cortex posterior (SOM – DMN)
	403	0.010 (0.004)	Inferior occipital gyrus - Superior temporal gyrus LH (VIS – SOM)
Biological sex	13288	0.040 (0.015)	Superior temporal gyrus medial - Heschl’s gyrus (SOM – SOM)
	12105	0.032 (0.011)	Cerebellum Crus II - Pars opercularis pars triangularis LH (Cerebellum - DMN)
	26166	0.030 (0.015)	Dorsomedial prefrontal cortex posterior - Frontal pole superior (DMN – DMN)
	27007	0.025 (0.012)	Pars opercularis - Inferior temporal sulcus posterior LH (VAN – DAN)
	1178	0.023 (0.011)	Lingual gyrus mid-anterior - Superior Parietal Lobule(HO) (VIS – DAN)
	14366	0.020 (0.007)	Temporal pole - Middle frontal gyrus anterior RH (Limbic – FPCN)
	21600	0.019 (0.008)	Superior parietal lobule middle - Supramarginal gyrus (DAN – VAN)
	2726	0.019 (0.009)	Insula - Superior temporal gyrus LH (VAN – SOM)
	29374	0.017 (0.007)	Anterior horizontal ramus lateral fissure RH - Middle frontal gyrus anterior (VAN – FPCN)
	21670	0.017 (0.007)	Superior parietal lobule middle - Cerebellum Crus I posterior (DAN - /)
	27144	0.015 (0.007)	Cerebellum VI anterior - Collateral sulcus middle (Cerebellum - VIS)
	15988	0.015 (0.007)	Cerebellum Crus II - Caudate superior (no network)
	12193	0.014 (0.006)	Cerebellum Crus II - Frontal pole inferior (Cerebellum - Limbic)
	32349	0.013 (0.004)	Superior longitudinal fasciculus I LH - Paracentral lobule (Cerebellum - SOM)

*Note.* PFI score = Permutation feature importance score; DiFuMo = Dictionary of Functional Modes for brain imaging; ROI = Region of interest; RH = right hemisphere; LH = left hemisphere; FPCN = frontopartietal central network; SOM = somatomotor network; DAN = Dorsal attention network; VIS = Visual network; DMN = Default mode network; VAN = Ventral attention network; HO = Harvard Oxford atlas

**Table 2.** Significant functional connections for education phenotype.

Feature	PFI score (std)	DiFuMo atlas ROI
16108	0.018 (0.006)	Cerebellum Crus II - Posterior cingulate (Cerebellum - DMN)
27455	0.017 (0.008)	Superior occipital sulcus LH - Lingual gyrus medial (DAN – VIS)
6404	0.015 (0.005)	Lateral occipital cortex anterior - Cuneus posterior (VIS – VIS)
5753	0.012 (0.005)	Occipital pole middle - Central sulcus inferior (VIS – SOM)
5520	0.009 (0.004)	Putamen anterior - Frontal pole superior (Cerebellum - DMN)
29008	0.009 (0.003)	Precuneus posterior - Transverse occipital sulcus (FPCN – VIS)
1910	0.009 (0.004)	Lingual gyrus middle - Frontal operculum LH (VIS – VAN)
8160	0.009 (0.004)	Parieto-occipital sulcus mid-anterior - Cerebellum IX (VIS - Cerebellum)
23825	0.008 (0.003)	Precuneus inferior - Precuneus inferior (DMN - Cerebellum)

*Note.* PFI score = Permutation feature importance score; DiFuMo = Dictionary of Functional Modes for brain imaging; ROI = Region of interest; RH = right hemisphere; LH = left hemisphere; FPCN = frontopartietal central network; SOM = somatomotor network; DAN = Dorsal attention network; VIS = Visual network; DMN = Default mode network; VAN = Ventral attention network

**Table 3.** Significant functional connections for handedness, race and number of languages phenotypes.

Phenotype	Feature	PFI score (std)	DiFuMo atlas ROI
Handedness	32457	0.010 (0.000)	Parietal operculum RH - Supramarginal gyrus (SOM – VAN)
	17838	0.008 (0.003)	Superior parietal lobule anterior - Supramarginal gyrus (DAN – VAN)
	22483	0.008 (0.004)	Inferior temporal sulcus posterior LH - Intraparietal sulcus inferior RH (DAN – FPCN)
Race	2015	0.011 (0.006)	Intraparietal sulcus superior RH - Intraparietal sulcus superior RH (DAN – DAN)
	31625	0.010 (0.005)	Supramarginal gyrus anterior LH - Supramarginal gyrus anterior LH (VAN – VAN)
	3940	0.010 (0.004)	Transverse occipital sulcus - Occipital pole inferior (VIS – VIS)
Number of languages	12672	0.009 (0.003)	Inferior occipital sulcus - Inferior occipital gyrus posterior (VIS – VIS)

*Note.* PFI score = Permutation feature importance score; DiFuMo = Dictionary of Functional Modes for brain imaging; ROI = Region of interest; RH = right hemisphere; LH = left hemisphere; FPCN = frontopartietal central network; SOM = somatomotor network; DAN = Dorsal attention network; VIS = Visual network; VAN = Ventral attention network;

**Table 4.** Significant functional connections for marital status and number of phenotypes.

Phenotype	Feature	PFI score (std)	DiFuMo atlas ROI
Marital status	20858	0.010 (0.000)	Planum temporale RH - Occipital Pole (SOM – VIS)
	5432	0.010 (0.000)	Frontal pole inferior - Cuneus posterior (Limbic – VIS)
	1609	0.009 (0.002)	Inferior frontal gyrus LH - Cingulate cortex mid-posterior (FPCN – VAN)
	18442	0.009 (0.002)	Middle frontal gyrus posterior RH - Occipital pole middle (FPCN – VIS)
	22594	0.009 (0.002)	Lingual gyrus anterior - Medial frontal cortex posterior (VIS – VAN)
	28812	0.009 (0.003)	Lateral occipital cortex inferior - Thalamus medial (VIS - Cerebellum)
	25365	0.008 (0.003)	Middle temporal gyrus middle RH - Angular gyrus superior (DMN – DMN)
	17261	0.008 (0.004)	Superior temporal sulcus RH - Inferior frontal gyrus LH (SOM – FPCN)
	2973	0.008 (0.004)	Cuneus posterior - Frontal operculum RH (VIS – SOM)
Number of children	28357	0.009 (0.002)	Calcarine sulcus anterior - Fusiform gyrus LH (VIS – VIS)
	7340	0.008 (0.003)	Dorsomedial prefrontal cortex - Cerebellum Crus I RH (FPCN - Cerebellum)
	28789	0.008 (0.003)	Lateral occipital cortex inferior - Insula anterior (VIS – VAN)

*Note.* PFI score = Permutation feature importance score; DiFuMo = Dictionary of Functional Modes for brain imaging; ROI = Region of interest; RH = right hemisphere; LH = left hemisphere; FPCN = frontopartietal central network; SOM = somatomotor network; DAN = Dorsal attention network; VIS = Visual network; DMN = Default mode network; VAN = Ventral attention network

**Table 5.** Significant functional connections for employment status phenotype.

Phenotype	Feature	PFI score (std)	DiFuMo atlas ROI
Employment status	9963	0.015 (0.005)	Inferior frontal gyrus anterior LH - Inferior frontal sulcus anterior LH (FPCN – FPCN)
	28497	0.010 (0.000)	Precentral gyrus middle - Inferior frontal gyrus LH (SOM – FPCN)
	16893	0.010 (0.000)	Cuneus superior - Caudate superior (VIS – Cerebellum)
	2080	0.009 (0.002)	Precuneus anterior - Middle frontal gyrus anterior LH (VAN – VAN)
	4383	0.009 (0.003)	Inferior frontal sulcus anterior LH - Cingulate cortex mid-anterior (FPCN – VAN)
	30995	0.009 (0.003)	Forceps major RH - Lateral occipital cortex superior (Cerebellum - DAN)
	17332	0.009 (0.003)	Superior temporal sulcus RH - Parieto-occipital sulcus mid-anterior (SOM – VIS)
	7654	0.008 (0.003)	Intraparietal sulcus posterior RH - Angular gyrus superior RH (DAN – FPCN)
	1824	0.008 (0.003)	Genu of callosal body - Corona radiata anterior (No networks)
	9224	0.008 (0.003)	Middle Frontal Gyrus(HO) - Superior frontal sulcus LH (DMN – DMN)
	23163	0.008 (0.003)	Superior parietal lobule posterior - Inferior occipital sulcus (DAN – VIS)
	24448	0.008 (0.004)	Precentral sulcus superior LH - Calcarine sulcus middle (DAN – VIS)

*Note.* PFI score = Permutation feature importance score; DiFuMo = Dictionary of Functional Modes for brain imaging; ROI = Region of interest; RH = right hemisphere; LH = left hemisphere; FPCN = frontopartietal central network; SOM = somatomotor network; DAN = Dorsal attention network; VIS = Visual network; DMN = Default mode network; VAN = Ventral attention network; HO = Harvard Oxford atlas

**Table 6.** Phenotypes and their classification

Phenotype	Classes (#)
Age	Young old (60–79 years); Old old (80–100+) (2)
Biological sex	Man; Woman (2)
Years of education	Middle (7–15 years); High (16–18); Very high (19–21) (3)
Handedness	Right (40–100); Mixed (−35–35); Left (−40– −100) (3)
Race	White Caucasian; African American; Asian (3)
Languages spoken	Only mother tongue; Two languages; Three languages or more (3)
Marital status	Single, not married; Married, in relationship; Divorced, separated, widowed (3)
Number of children	None; 1 or 2 children; 3 or more children (3)
Employment status	Employed; Not employed (2)
